# Serological screening of immunoglobulin M and immunoglobulin G during pregnancy for predicting congenital cytomegalovirus infection

**DOI:** 10.1186/s12884-019-2360-1

**Published:** 2019-06-20

**Authors:** Yuka Torii, Shigeru Yoshida, Yoichiro Yanase, Takashi Mitsui, Kazuhiro Horiba, Toshihiko Okumura, Suguru Takeuchi, Takako Suzuki, Jun-ichi Kawada, Tomomi Kotani, Mamoru Yamashita, Yoshinori Ito

**Affiliations:** 1 Department of Pediatrics, Nagoya University Graduate School of Medicine, 65 Tsurumai-cho, Showa-ku, Nagoya, 466-8550 Japan; 2 Department of Pediatrics, Kishokai Medical Corporation, 4-122 Koike, Inazawa, 492-8144 Japan; 3 Department of Obstetrics and Gynecology, Kishokai Medical Corporation, 4-122 Koike, Inazawa, 492-8144 Japan; 4 Department of Obstetrics and Gynecology, Nagoya University Graduate School of Medicine, 65 Tsurumai-cho, Showa-ku, Nagoya, 466-8550 Japan

**Keywords:** Serological screening, Immunoglobulin M, Immunoglobulin G, Congenital cytomegalovirus infection

## Abstract

**Background:**

Cytomegalovirus (CMV) is one of the most frequent pathogens for congenital infections. Most cases of congenital CMV infection (cCMV) are asymptomatic at birth, but sensorineural hearing loss (SNHL) or neurodevelopmental delay can appear later in childhood. This prospective study examined the practicability of serological screening for anti-CMV immunoglobulin (Ig) G and anti-CMV IgM in pregnant women.

**Methods:**

A total of 11,753 pregnant women were examined for CMV IgG and CMV IgM during the first or second trimester. When IgM was positive, IgG was reevaluated more than two weeks later. When IgG was negative, IgG was reevaluated in the second or third trimester. All neonates from mothers with positive/borderline IgM or IgG seroconversion underwent polymerase chain reaction assay for CMV using urine samples to diagnose cCMV. Levels of IgG and IgM were compared between mothers with and without cCMV. Receiver operating characteristic (ROC) curves for IgM titers were analyzed.

**Results:**

Eight of 500 neonates (1.6%) born from mothers with positive IgG and positive IgM, and 3 of 13 neonates (23.1%) born from mothers with IgG seroconversion were diagnosed with cCMV. Neither IgM titers nor IgG titers differed significantly between cCMV and non-cCMV groups. The area under the ROC curve was 0.716 and the optimal cut-off for IgM was 7.28 index (sensitivity = 0.625, specificity = 0.965, positive predictive value = 0.238, negative predictive value = 0.993). Titers of IgG were not frequently elevated in pregnant women with positive IgM during the observation period, including in those with cCMV. All 11 cCMV cases were asymptomatic at birth and none had shown SNHL or developmental delay as of the last regular visit (mean age, 40 months).

**Conclusions:**

Seroconversion of CMV IgG and high-titer IgM during early pregnancy are predictors of cCMV. High IgM titer (> 7.28 index) is a predictor despite relatively low sensitivity. Levels of IgG had already plateaued at first evaluation in mothers with cCMV. Maternal screening offered insufficient positive predictive value for diagnosing cCMV, but allowed identifying asymptomatic cCMV cases in an early stage.

## Background

Cytomegalovirus (CMV) is one of the most common pathogens in perinatal infection. The incidence of congenital CMV infection (cCMV) is 1 in 300 live births in Japan, where the CMV seroprevalence is about 70% [1, 2]. Most neonates with cCMV are born without physical symptoms, with around 10–15% of cCMV cases showing physical symptoms [3]. The clinical manifestations of cCMV are divided into early findings that appear at birth, and late findings that do not appear until later in childhood [4]. Early findings of cCMV include small for gestational age, microcephaly, petechiae, jaundice, hepatosplenomegaly, and purpura. Intracranial calcification, periventricular cyst or ventriculomegaly, sensorineural hearing loss (SNHL), and retinitis can be detected by examinations. Late findings such as developmental delay can appear as the patient matures. SNHL can also become apparent as a later symptom [5]. Utilization of real-time polymerase chain reaction (PCR) assays has expanded and this is now the standard procedure for diagnosing cCMV [6, 7]. The effectiveness of ganciclovir therapy in preventing hearing deterioration was reported in 2003 [8]. More recently, a 6-month protocol of oral valganciclovir improved hearing and neurodevelopment at the long-term assessment compared to the 6-week protocol [9].

Primary CMV infection during pregnancy has been known to carry a high risk of fetal infection, and hygiene education for CMV-seronegative pregnant women is efficient for preventing cCMV [10]. As cCMV is becoming better recognized in the general population, the number of pregnant women requesting maternal screening for primary CMV infection is increasing. However, implementation of routine maternal screening for primary CMV infection remains somewhat contentious due to several issues [3]. Firstly, definitive diagnosis of primary CMV infection using serological tests alone is difficult. A positive result for immunoglobulin (Ig) M is difficult to diagnose as primary infection, because CMV IgM persists for a long time (> 5 months to over 1 year) after infection in some individuals (known as “persistent IgM”) [11, 12]. The clinical cut-off titer for IgM has not been determined and may need further investigation. IgG avidity may provide an accurate indicator of recent primary infection [12, 13], but the IgG avidity test is labor-intensive and the commercial availability is limited. Secondly, not a small number of cCMV neonates are born from mothers with non-primary infection [14–16]. Because the usefulness of maternal screening for cCMV has not been confirmed, we conducted the present prospective study to examine the practicability of serological screening for anti-CMV IgG and anti-CMV IgM in pregnant women.

## Methods

### Study design and participants

This prospective study of a nested cohort was conducted between April 2014 and February 2017. A total of 11,753 pregnant women who underwent a first CMV screening test at any of 12 clinics in Aichi and Gifu prefectures in Japan were included in the study. Inclusion criteria were as follows: 1) pregnant woman capable of providing written consent for the study; and 2) screening performed using CMV IgG and CMV IgM during the first and second trimesters. Exclusion criteria were: 1) spontaneous abortion; 2) unavailability of a neonatal urine sample, e.g., moving. Pregnant women with negative results for both IgG and IgM were advised regarding hygiene methods to prevent primary CMV infection. IgG was then reevaluated at 22 weeks of gestation or later. Neonates from pregnant women with IgG seroconversion were subjected to CMV PCR using urine samples. Pregnant women with positive IgG and negative IgM were considered as having had previous CMV infection, and no further investigation was performed. Pregnant women with positive/negative IgG and positive/borderline IgM at first screening were reevaluated for IgG after more than 2 weeks. Neonates from pregnant women with positive/negative IgG and positive/borderline IgM were also subjected to CMV PCR. Expecting mothers were also asked to report any cold-like symptoms, such as fever, chills, sore throat, rhinorrhea or cough, during the intervals between regular visits. All study protocols were approved by the research ethics committee at Nagoya University (permission number: 2017–0126).

### Serological tests for CMV

Serological tests for CMV IgM and IgG were performed using enzyme immunoassay (EIA) kits (Denka Seiken, Tokyo, Japan) or chemiluminescent immunoassay (CLIA) kits (Abbott Japan, Matsudo, Japan). Threshold levels were determined using the manufacturer’s protocols. For EIA kits, thresholds were defined as follows: CMV IgM (negative, < 0.8; borderline, 0.8 to < 1.2; positive, ≥1.2 index), CMV IgG (negative, < 2; borderline, 2 to < 4; positive, ≥4 EIA titer). For CLIA kits, thresholds were defined as follows: CMV IgM (negative, < 0.85; borderline, 0.85 to < 1.0; positive, ≥1.0 index), CMV IgG (negative, < 8; positive, ≥8 CLIA titer).

### PCR assay for CMV

Urine samples from neonates were obtained within 3 days after birth. The urine samples were stored at − 30 °C or immediately used for DNA extraction. DNA extraction was performed using the QIAamp Viral RNA Mini Kit (Qiagen, Hilden, Germany). The kit is optimized for viral RNA extraction but can also extract viral DNA. Extracted DNA was analyzed by real-time PCR to detect CMV DNA, as described previously with a slight modification [17]. In brief, assays were performed using Quantstudio 3 (Applied Biosystems, Foster City, CA), in a total volume of 25 μl consisting of 12.5 μl Taqman Fast Advanced Mix (Applied Biosystems), 0.05 μl each of 50 μM sense and antisense primers, 0.025 μl of 100 μM probe and 7.375 μl of nuclease free water.

### Follow-up patients with cCMV

When CMV PCR was positive, the baby was brought to Nagoya University Hospital for medical examination. The baby received a physical examination, neurological examination, audiological testing using the auditory brainstem response, ophthalmologic examination, brain ultrasound, and brain MRI for evaluation of cCMV. The neonates were diagnosed as having symptomatic cCMV if they had any of the following findings; small for gestational age, microcephaly, petechiae, jaundice, hepatosplenomegaly, purpura, intracranial calcification, periventricular cyst or ventriculomegaly, SNHL, or retinitis. Regular medical visits were continued for evaluation of hearing and development until 4 years old even if the baby did not have any symptoms.

### Statistical analysis

A data entry screen was created using Excel software (Microsoft, Redmond, WA). Data were checked, coded and entered into the computer. Data were double-checked. The following descriptive statistics were calculated: frequency, percent, mean and standard deviation. The incidence of cCMV was compared between the IgG seroconversion group and positive IgG/positive IgM group using the Fisher’s Exact test. Titers of CMV IgG and IgM by EIA at first screening were compared between cases with and without cCMV using the Mann-Whitney test. Receiver operating characteristic (ROC) curves of IgM titers were analyzed. Statistical analyses were performed using SPSS version 24.0 (IBM, Chicago, IL).

## Results

**Fig. 1:**
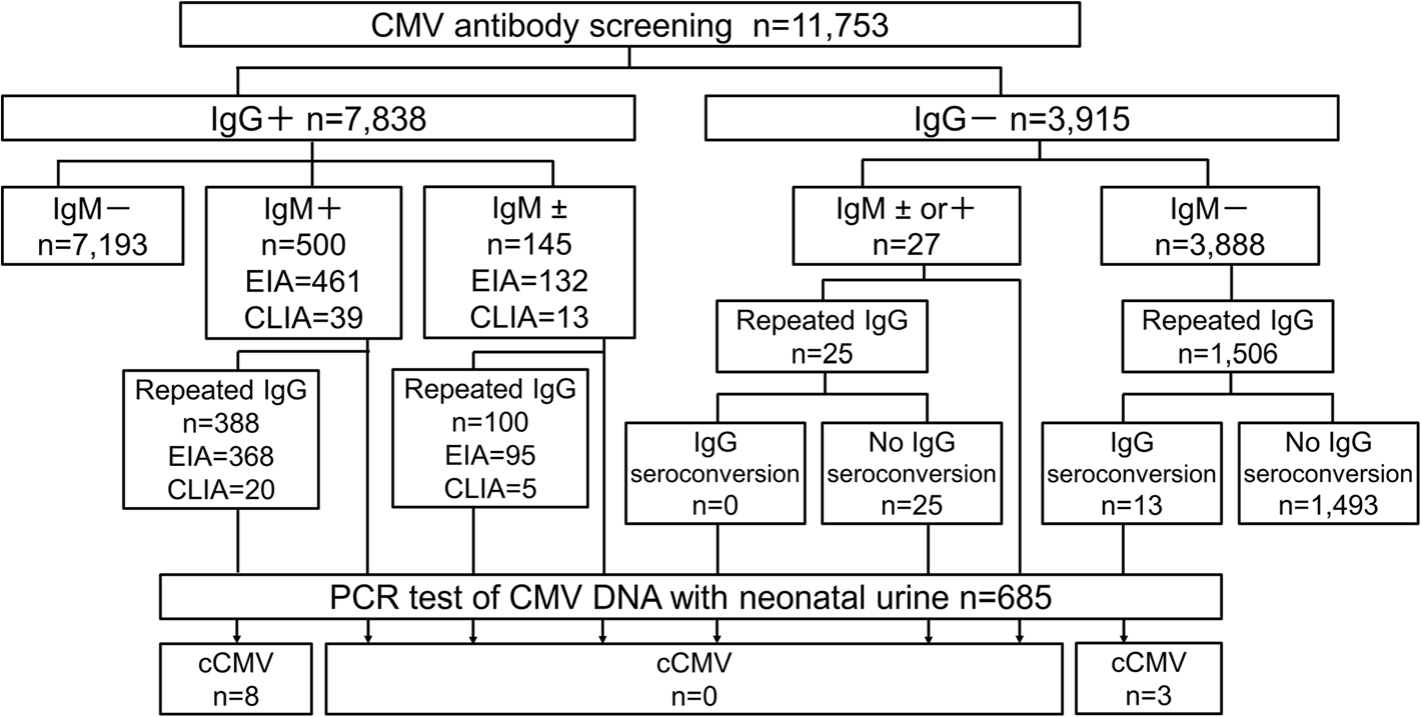
Flow diagram of study participants and results of serological screening. Results for the population that underwent serological screening. Neonates with borderline IgM born to positive mothers or IgG-seroconverted mothers (*n* = 685) underwent urine PCR assay to evaluate cCMV infection

The results of serological screening for pregnant women and PCR for neonates are shown in Fig. 1. Of the 685 newborns subjected to PCR, 11 newborns were diagnosed with cCMV. All 11 cCMV cases were asymptomatic at birth and none of them showed SNHL or developmental delay as of the last regular visit (mean age, 40 months). The clinical characteristics of pregnant women subjected to neonatal urine CMV PCR test are shown in Table 1.

**Table 1:** Clinical characteristics of cCMV and non-cCMV mothers

Characteristics	cCMV mothers	Non cCMV mothers	*P* value
	*n* = 11	*n* = 674	
Age (years)	31 (17–39)	32 (17–45)	0.29
Gestational weeks at delivery (weeks)	39.9 (36.6–41.1)	39.9 (36.0–42.0)	0.76
Gestational weeks at initial CMV antibody screening (weeks)	11.6 (9.7–13.9)	11.1 (3.3–26.3)	0.25
Cold-like syndrome during pregnancy	2 (18.2%)	92 (13.6%)	0.66

Quantitative data are expressed as median and range, and qualitative data are expressed as number and percentages

**Fig. 2:**
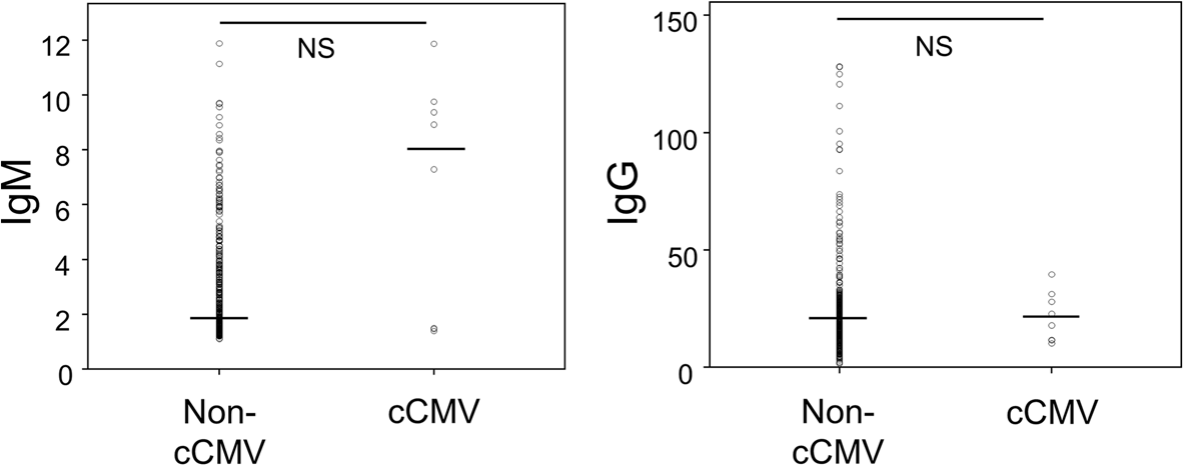
Dot plot for CMV IgG and IgM titers in the cCMV and non-cCMV groups. Maternal IgM titers were compared between non-cCMV and cCMV groups. The short horizontal bar indicates the median (cCMV group, *n* = 8; non-cCMV group, *n* = 453)

**Fig. 3:**
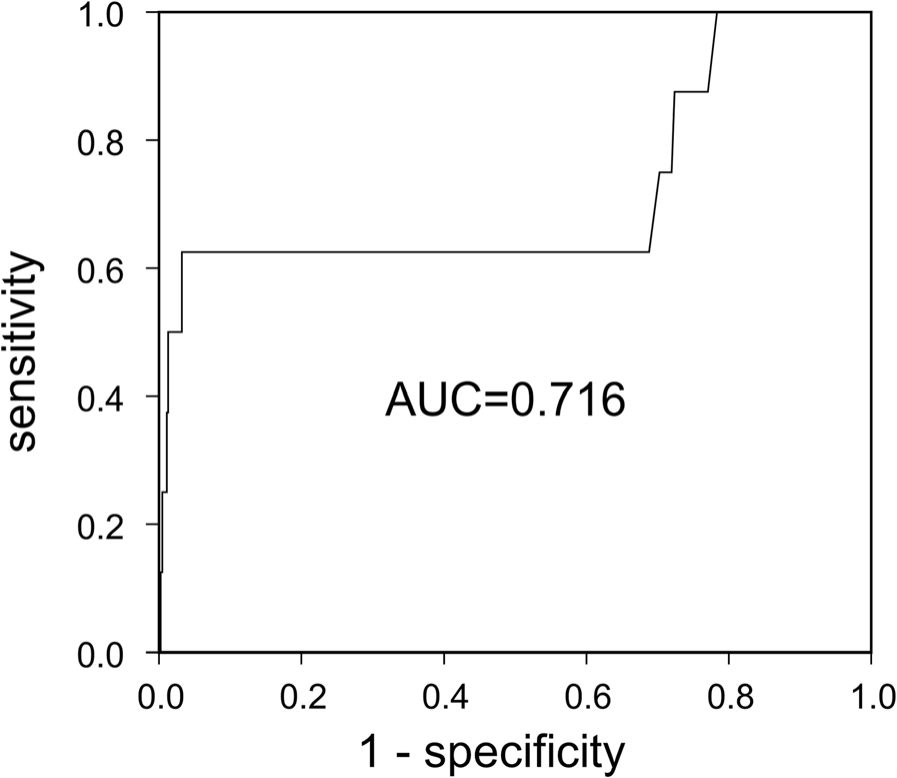
Receiver operating characteristic (ROC) curve for IgM titers in mothers with cCMV. ROC curve analysis was used to determine the diagnostic cutoff. Area under the ROC curve was 0.716, suggesting moderate usefulness of the IgM titer as a prognostic marker for cCMV. The optimal cut-off was 7.28 (sensitivity = 0.625, specificity = 0.965)

Maternal IgM and IgG titers (EIA) were compared between the non-cCMV group (*n* = 453) and cCMV group (*n* = 8) (Fig. 2). Neither IgM nor IgG titers differed significantly between the cCMV and non-cCMV groups. Next, an ROC curve was generated to assess the threshold level of IgM. The 9pt?>ROC curve showed that area under the curve was 0.716, suggesting the moderate usefulness of the titer of IgM as a prognostic marker for cCMV (optimal cut-off = 7.28 index, sensitivity = 0.625, specificity = 0.965, positive predictive value = 0.238, negative predictive value = 0.993) (Fig. 3).

**Fig. 4:**
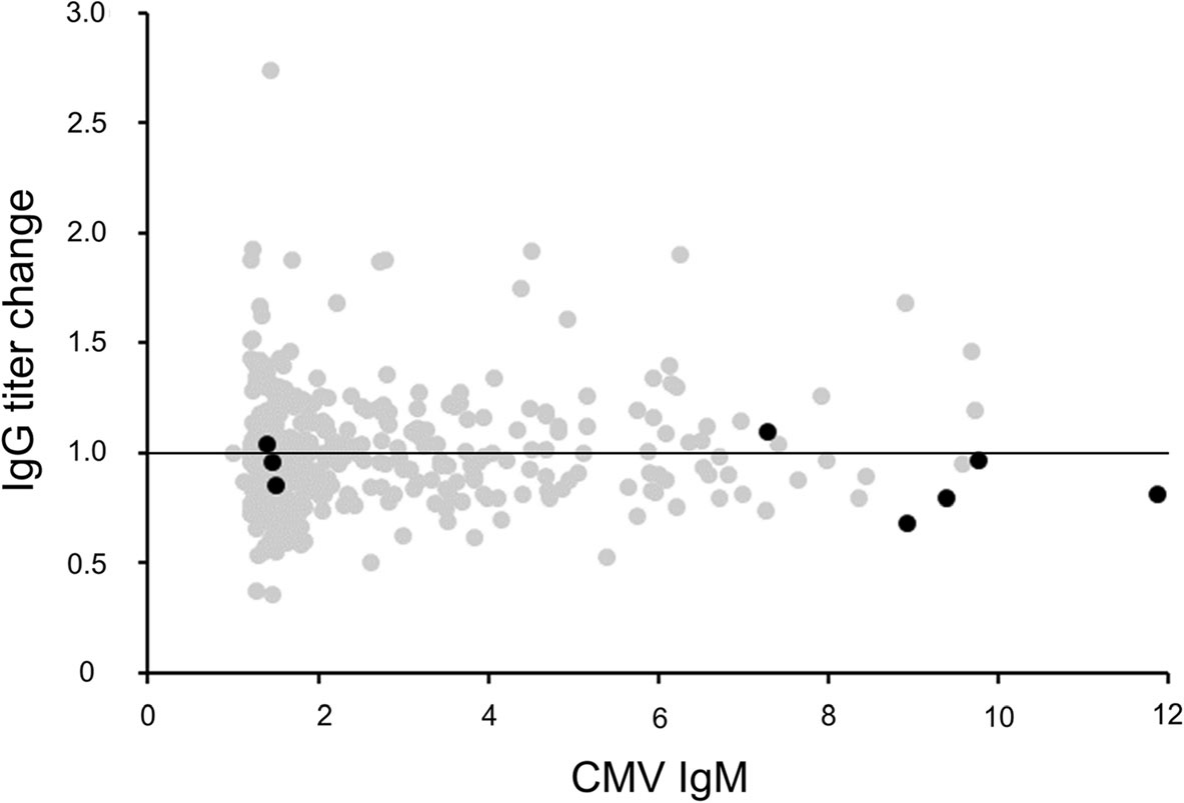
Scatter plot for CMV IgM titer and fold-change of CMV IgG. The fold change of IgG was determined as the titer of IgG at the second evaluation / titer of IgG at the first screening. The interval between blood samplings ranged from 12 to 50 days. Black dots indicate cases with cCMV and grey dots indicate cases without cCMV. Horizontal line indicates a ratio of 1.0

IgG titers were reevaluated more than 2 weeks later in those pregnant women who showed positive IgM at screening. The fold change in IgG (titer of IgG at second evaluation / titer of IgG at first screening) was determined. No correlations were seen between titer of IgM and titer change ratio of IgG. Interestingly, the ratio of titer change of IgG in mothers with cCMV ranged from 0.85 to 1.09, suggesting that levels of IgG had already plateaued by the first evaluation (Fig. 4).

## Discussion

Our data demonstrated the epidemiology of maternal CMV infection in the Chubu region of Japan. We examined more than 10,000 pregnant women and found that the seroprevalence of CMV was 66.7%, almost the same as previous reports from Japan (68.1% [2]; 69.1% [18]). About 0.09% of pregnant women shown to be IgG seronegative underwent seroconversion. The incidence of cCMV was significantly higher in mothers with IgG seroconversion (23.1%) than in IgG-positive, IgM-positive mothers (1.6%) (*p* = 0.002). Positive/borderline IgM together with negative IgG didn’t show the risk for cCMV infection.

Screening exclusively identified pregnant women with IgG seronegative or primary infection as insufficient for predicting cCMV. Increasing evidence suggests that a not insubstantial number of symptomatic cCMV cases are born from non-primary infected mothers, and are thus quite difficult to diagnose from serological examination [14, 19]. Ross at al. reported that 29% of healthy women with CMV IgG seropositive results showed reinfection with CMV during the study period of 3 years [20]. Picone et al. reported the difficulty for non-primary CMV infection showing various serological statuses of mothers with cCMV [21]; among 9 cases with non-primary infection, titers of IgG increased in 3 cases, and two showed positive IgM. IgG avidity decreased in 1 case, despite positive IgM. In the present study, IgG avidity index was not evaluated in pregnant women with positive IgM, because the commercial availability of IgG avidity index was limited. Another limitation was that the serostatus before pregnancy was not evaluated in enrolled women.

We investigated IgG titer changes in pregnant women with positive IgM to confirm primary CMV infection. The IgG titer was not frequently increased in cases with positive IgM, including in mothers with cCMV. Titers of IgM did not appear to correlate with increases of IgG during the observation period. The reason is that the paired sera assay is generally used for diagnosis of acute viral infection, but is unsuitable for CMV screening because pregnant women are often asymptomatic in CMV infection and the timing of sampling sera may be inappropriate. Regarding the results of IgM, the titer of IgM in cases with cCMV was not significantly different compared to those without cCMV in the present study. A high titer of IgM (> 7.28 index) is a predictor despite the relatively low sensitivity. Toriyabe et al. reported high-titer IgM with low IgG avidity index as a predictor of cCMV [22]. Dollard et al. reported high-titer IgM as a strong predictor of a low index of IgG avidity, suggesting primary infection [23]. In the present study, none of the cases with negative IgG and borderline/positive IgM showed IgG seroconversion. Interestingly, titers of IgM were relatively low in most of these cases. These results of IgM are therefore thought to represent false positives. A previous report also demonstrated no IgG seroconversions in pregnant women with negative IgG/positive IgM (*n* = 57) [22].

In our study, all confirmed cCMV neonates were asymptomatic at birth. Asymptomatic cCMV born from IgG-seroconverted mothers may be due to CMV infection in the relatively late term of pregnancy. Those mothers were confirmed as IgG-seronegative at 9.7–12.0 weeks of gestational age and seroconversion was observed at a gestational age of 24–36 weeks. Enders et al. reported that the rate of intrauterine transmission was higher in the third trimester (72%) than in the first trimester (30.1%), but symptoms were more severe in neonates infected during early pregnancy [24]. The incidence of symptomatic neonates in total cCMV is about 11% [25, 26] to 30% [1].

Another controversial issue in universal screening for maternal CMV infection is how to manage asymptomatic patients who have potential for late-onset SNHL and developmental delay. All cCMV neonates have so far shown no neurological sequela in the present study. However, some proportion of children develop SNHL despite showing asymptomatic cCMV at birth [27–29]. Although antiviral therapy may prevent development of SNHL for such children with cCMV, evidence for the efficacy of this treatment remains lacking [30]. A clinical trial evaluating valganciclovir treatment to prevent development of SNHL in infants with asymptomatic cCMV is being performed by the National Institute of Allergy and Infectious Diseases [31]. Another issue to be resolved is to find biomarkers for potential late-onset symptoms in asymptomatic cCMV. Neonatal blood or urine CMV load may be useful for predicting late-onset symptoms [32–34]. Moreover, a clinical trial of ganciclovir therapy for mothers with CMV infection is expected to improve fetal outcomes [35], and another trial for CMV-specific hyperimmune globulin is also ongoing [31].

## Conclusions

In conclusion, seroconversion of CMV IgG is predictive of cCMV. High-titer IgM is a predictor, despite the relatively low sensitivity. Levels of IgG had already plateaued at first evaluation in cases with cCMV. Follow-up strategies are required for not only symptomatic cCMV, but also asymptomatic cCMV. Although universal screening for all pregnant women to assist in the diagnosis of cCMV is not currently recommended, asymptomatic cCMV is difficult to diagnose without universal screening for cCMV, as some proportion of patients develops late-onset SNHL and developmental delay. From our study, providing maternal screening offered insufficient positive predictive value for diagnosing cCMV, but allowed identifying asymptomatic cCMV cases in an early stage.

## Data Availability

The dataset used and/or analyzed during the present study is available from the corresponding author on reasonable request.

## Abbreviations

cCMV: Congenital CMV infection
CLIA: Chemiluminescent immunoassay
CMV: Cytomegalovirus
EIA: Enzyme immunoassay
IgG: Immunoglobulin G
IgM: Immunoglobulin M
PCR: Polymerase chain reaction
ROC: Receiver operating characteristic
SNHL: Sensorineural hearing loss
